# A Versatile Vector for *In Vivo* Monitoring of Type I Interferon Induction and Signaling

**DOI:** 10.1371/journal.pone.0152031

**Published:** 2016-03-23

**Authors:** Estanislao Nistal-Villan, Joanna Poutou, Estefania Rodríguez-Garcia, Maria Buñuales, Beatriz Carte-Abad, Jesus Prieto, Gloria Gonzalez-Aseguinolaza, Ruben Hernandez-Alcoceba, Esther Larrea

**Affiliations:** 1 Gene Therapy and Regulation of Gene Expression Program, Center for Applied Medical Research (CIMA), University of Navarra, Pamplona, Spain; 2 Instituto de Salud Tropical, University of Navarra, Pamplona, Spain; 3 IdiSNA Navarra Institute for Health Research, Pamplona, Spain; University of Geneva, SWITZERLAND

## Abstract

Development of reporter systems for *in vivo* examination of IFN-β induction or signaling of type I interferon (IFN-I) pathways is of great interest in order to characterize biological responses to different inducers such as viral infections. Several reporter mice have been developed to monitor the induction of both pathways in response to different agonists. However, alternative strategies that do not require transgenic mice breeding have to date not been reported. In addition, detection of these pathways *in vivo* in animal species other than mice has not yet been addressed. Herein we describe a simple method based on the use of an adeno-associated viral vector (AAV8-3xIRF-ISRE-Luc) containing an IFN-β induction and signaling-sensitive promoter sequence controlling the expression of the reporter gene luciferase. This vector is valid for monitoring IFN-I responses *in vivo* elicited by diverse stimuli in different organs. Intravenous administration of the vector in C57BL/6 mice and Syrian hamsters was able to detect activation of the IFN pathway in the liver upon systemic treatment with different pro-inflammatory agents and infection with Newcastle disease virus (NDV). In addition, intranasal instillation of AAV8-3xIRF-ISRE-Luc showed a rapid and transient IFN-I response in the respiratory tract of mice infected with the influenza A/PR8/34 virus lacking the NS1 protein. In comparison, this response was delayed and exacerbated in mice infected with influenza A/PR/8 wild type virus. In conclusion, the AAV8-3xIRF-ISRE-Luc vector offers the possibility of detecting IFN-I activation in response to different stimuli and in different animal models with no need for reporter transgenic animals.

## Introduction

The interferon (IFN)-β induction pathway and type I IFN (IFN-I) signaling are two related pathways culminating in the induction of critical antiviral and immuno-stimulatory genes [1]. The IFN-β induction pathway activates IFN regulatory factor (IRF) 3 and 7, which can bind specific IRF genomic DNA elements called IRF-E and stimulate the transcription of several genes [2]. Type I IFNs, including IFN-β, bind IFN-I receptor and trigger the IFN-I signaling cascade, activating STAT1, STAT2 and IRF-9. These three transcription factors form the so-called IFN-stimulated gene factor 3 (ISGF3) complex. ISGF3 binds DNA elements named IFN stimulated response elements or ISRE [3], triggering transcription of IFN-stimulated genes (ISGs) and the subsequent activation of cellular pathways associated with IFN stimulation.

IRF-E elements present a consensus sequence: WWNNRAAANNGAAA) [4], where W can be A/T, R can be G/A and N any nucleotide. The ISRE consensus sequence is GAAANNGAAACT [5]. The similarity of both consensus sequences supports the fact that many genes can be activated by both signaling pathways, such as *Mx2* [6] or *ISG15* [7]. Schmidt et al. [7] completed a thorough manipulation of ISG15 IRF-E and ISRE elements and found a sequence with optimized IRF-7 and ISGF3 binding properties (TCGGGAAACCGAAACT). This sequence is named the IRF-ISRE element throughout this text.

Several groups have generated transgenic mice expressing reporter genes under the control of IFN-β [8,9], IFN-α6 [10] or Mx2 promoters [6] to detect IFN induction or IFN-I activity *in vivo*. In addition, the IFN-specific responsive promoter Mx1 has been used to control the expression of Cre recombinase in mice [11] in order to generate conditional knock out models.

All reporter systems for IFN-β induction or type I IFN signaling described to date require the use of animal transgenesis and husbandry. These methods are expensive, time-consuming and restricted to a particular mouse strain. In addition, many viral infections have other animal models different from mice. Monitoring IFN-I responses *in vivo* in these other animal species is not an option, and detection of the IFN-I signature requires other invasive methods.

In the present study, we explored the possibility of developing an adeno-associated virus (AAV) reporter vector which enables live monitoring of IFN-I signature in different organs and animal species. AAV is a small, nonpathogenic parvovirus extensively used in gene transfer approaches. AAV-based vectors allow long-term expression *in vivo* without virus replication, and can transduce different organs/tissues depending on the serotype and/or the route of administration [12]. AAV vectors based on serotype 8 (rAAV8) can transduce the liver with high efficiency [13] when injected intravenously (iv) and the upper respiratory tract when inoculated through the nasal route [14]. The ability of this vector to deliver inducible expression systems has been previously demonstrated [15].

We describe here an AAV vector carrying an IRF-ISRE inducible sequence that controls the expression of firefly luciferase reporter gene (AAV8-3xIRF-ISRE-Luc). We have tested its ability to respond to different stimuli in different organs in C57BL/6 mice and in the liver of Syrian Hamsters.

## Materials and Methods

### Cells lines

The human cell lines HuH-7 (JCRB Genebank, Japan), Hep2 (ATCC CCL-23) and HepG2 (ATCC HB-8065), mouse cells Hepa1.6 (ATCC CRL-1830) and B16-OVA (courtesy of Dr. P. Sarobe, CIMA, Spain) [16] and the Syrian hamster cell line H2T (courtesy of Dr. C.M. Townsend, University of Texas, Galveston, TX, USA) [17] were cultured in Dulbecco’s modified Eagle’s medium supplemented with 10% heat-inactivated fetal bovine serum, 2 mM L-glutamine, 50 μg/ml penicillin/streptomycin (all culture reagents from Invitrogen). All cells were grown at 37°C in a 5% CO_2_ incubator.

### Reagents

The following reagents were used throughout the experiments performed *in vitro* or *in vivo*: human IFN-α (Sicor Biotech), human IFN-β, mouse IFN-α and mouse IFN-β (PBL). R848 (InvivoGen), CpG and DEAE Dextran (Sigma) and poly I:C (Oncovir).

### Reporter assays

All cell culture reporter assays were performed using the indicated IFN-β induction or IFN-I signaling sensitive DNA elements cloned into the pGL4.17 Firefly luciferase reporter plasmid (Promega). pRL-TK plasmid was used in all cases in order to normalize pGL4.17 reporter plasmid induction. Cell transfection with the appropriate plasmids was performed as described [18]. A dual-luciferase reporter assay kit (Promega) was used to measure reporter activities following the manufacturer’s instructions.

### Plasmid construction

Plasmids containing artificial IRF-ISRE or MxA ISRE sequences fused to a minimal MxA promoter sequence were synthesized by GenScript. The regions of interest were subcloned into a luciferase reporter plasmid pGL4.17[*luc2*/Neo]. Reporter plasmids bearing multiple copies of IRF-ISRE were obtained by inserting a DNA element composed of two complementary oligonucleotide sequences containing the IRF-ISRE element. This insert was flanked by PacI restriction sites that were used to insert multiple repetitions of this insert into a PacI site between the original IRF-ISRE and MxA promoter sequences. AAV-MCS vector (Cell Biolabs) was used to insert the promoter and reporter elements. CMV promoter was removed and the 3xIRF-ISRE-MxA-luciferase sequence was subcloned into the AAV multi cloning site.

### Viruses

AAV8-3xIRF-ISRE-Luc rescue was performed as described previously [19]. Recombinant rNDV-F3aa-GFP LaSota, Sendai Cantell virus and influenza viruses used to infect mice and hamsters have been described before [14]. Viral doses per animal are expressed as viral genomes (vg) for AAV vectors and infectious units (iu) for NDV and influenza viruses.

### Animal handling and treatments

Six week old female C57BL/6 mice and Syrian (Golden) hamsters (*Mesocricetus Auratus*; HSD HAN: AURA, 5 weeks of age) were purchased from Harlan Laboratories. Animals were maintained under pathogen-free conditions in the CIMA’s animal facility. All of the animal procedures were performed in accordance with institutional guidelines and were approved by the Animal Experimentation Ethics Committee of the University of Navarra (Permit number: CEEA-153-14). All animal manipulations were performed under isofluorane inhalatory anesthesia (Abbott labs). Hydrodynamic plasmid injection in mice was performed as previously described [20], using 20 μg of reporter plasmids. IFN-β was administered intraperitoneally at the indicated doses. The IFN-agonists: poly I:C (50 μg), CpG DNA (50 μg) and imiquimod-R848 (100 μg) were administered intravenously. For intranasal administration, viruses were formulated in 50 μl saline solution and instilled drop by drop during at least 1 minute in anesthetized animals.

### Bioluminescence imaging (BLI)

For in vivo quantification of luciferase activity in animals transduced with the AAV8-3xIRF-ISRE-Luc vector, mice and hamsters were anesthetized and received an intraperitoneal injection of 100 and 300 μl D-luciferin potassium-salt substrate (Promega), respectively, dissolved in PBS at a final concentration of 30 mg/ml. Light emission was measured using a Photon Imager device (Biospace Lab). Photon counts were acquired 8 minutes after substrate administration during 1 minute. Light surface images were obtained immediately after each photon counting session to provide an anatomical view of the animal. Image processing and signal intensity quantifications were performed using M3 Vision software (Biospace Lab). Images are displayed as a pseudo-color photon count image, superimposed on a gray scale anatomic white-light image, allowing assessment of both bioluminescence intensity and its anatomical source. The number of photons emitted per second per square centimeter per steradian was calculated as a measure of luciferase activity utilizing a constant region of interest.

### Analysis of gene expression

Peripheral blood mononuclear cells were purified using Ficoll-Paque (GE Bioscience). Total RNA from cells or liver tissue was extracted using the automated MagMax Express 96 system (Applied Biosystems) using the Magmax-96 total RNA isolation kit (Life Technologies). Reverse transcription was performed as previously reported [21]. Real-time polymerase chain reactions were performed with iQ SYBR Green supermix (Bio-Rad) in a CFX96 system from Bio-Rad, using specific primers for each gene [14]. The amount of each transcript was expressed by the formula: 2^ct(β-actin)−ct(gene)^, with ct being the point at which the fluorescence rises appreciably above the background fluorescence.

### Quantification of neutralizing antibodies

The reporter virus NDV-GFP was serially diluted in a 1:2 series. A virus dilution corresponding to the last dose presenting near 100% GFP signal in Hep2 cells was later used for incubation at 25°C for 2 hours with mouse or hamster sera. Ab neutralizing titers were obtained in quadruplicate using the Reed-Muench method to calculate the inhibitory ED50 also in Hep2 cells.

### Measurement of type I IFNs by bioassay and ELISA

The amount of type I IFN present in serum from hamsters was analyzed by measuring its ability to protect hamster H2T cells against the cytopathic effect of encephalomyocarditis virus. The assay was performed in a 96-well microtiter plate. First, 2x10^4^ H2T cells per well were seeded in 150 μl of medium containing serial dilutions of serum. After incubation for 24 hours, cells were infected with encephalomyocarditis virus (5x10^6^ pfu per well), and 24 hours later, the cytopathic effect was measured by staining with crystal violet dye solution (0.5% in 1/4 v/v methanol/water). The optical density was read at 595 nm. At the same time, serial dilutions of human IFN-α were tested to obtain a standard curve. Results are calculated interpolating the optical density of each sample in the standard curve and are expressed as units/ml.

The levels of IFN-α and IFN-β protein in serum samples from mice were quantified by ELISA (PBL assay science) following the manufacturer΄s instructions.

### Statistical analysis

Statistical analysis was performed using PRISM version 5.0 (GraphPad). Data are presented as mean ± SD. Comparisons between two groups were made using Mann Whitney or two-tailed unpaired t-test. Multiple groups were compared using ANOVA with Tukey’s post-test. Statistical significance was assigned to p-values less than 0.001 (***), less than 0.01 (**) or less than 0.05 (*).

## Results

### *In vitro* characterization of IFN-I reporters

**Fig 1:**
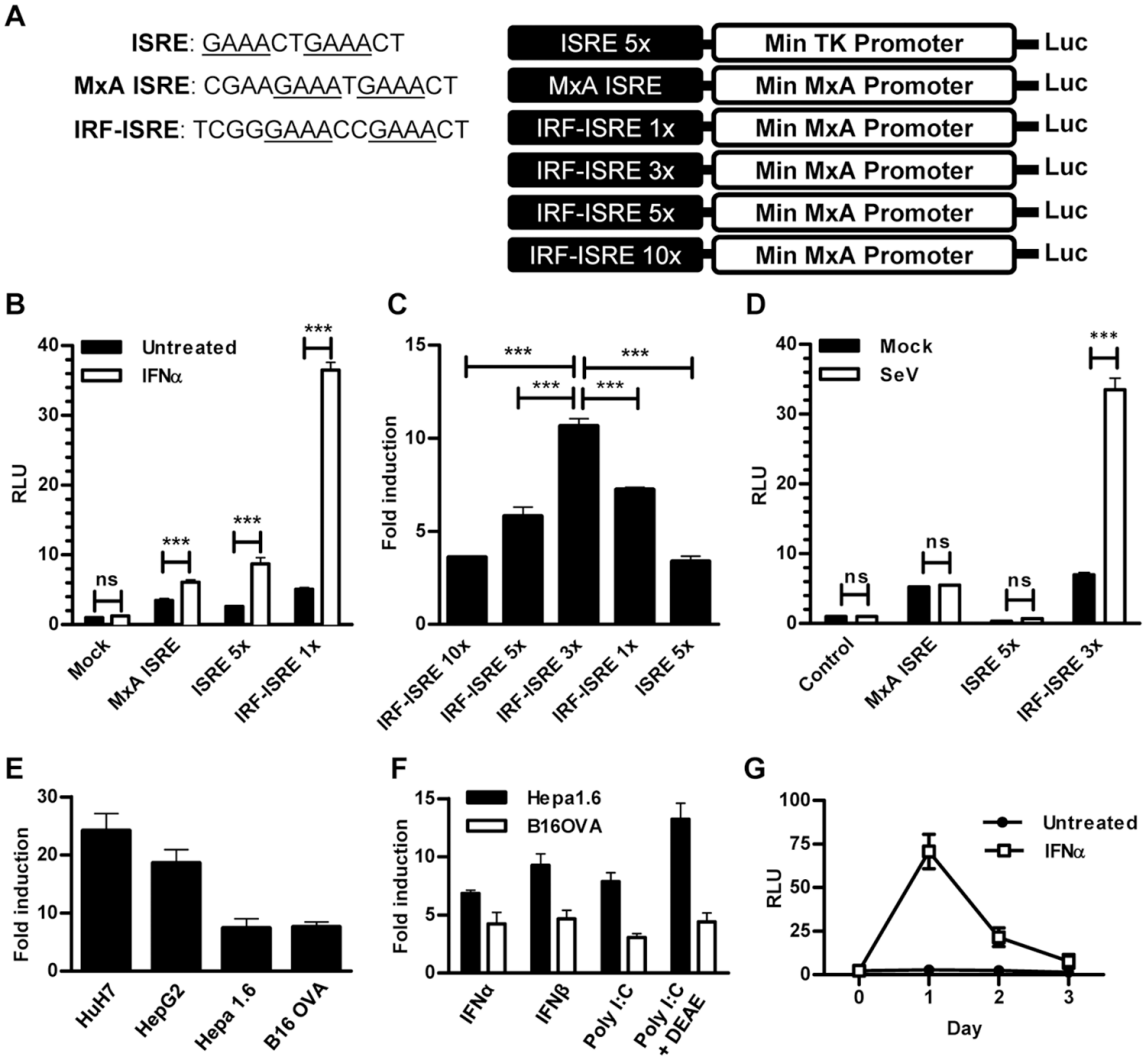
*In vitro* characterization of IFN-I reporters. A) Schematic representation of reporter plasmid constructs. B) Dual luciferase reporter assay in HuH-7 cells transfected with the indicated plasmids in response to 500 units/ml of human IFN-α for 24 hours. C) Luciferase activity (fold induction) in HuH-7 cells transfected with the indicated reporter plasmids and treated with 500 units/ml of human IFN-α for 24 hours. D) Dual luciferase reporter assay in Hepa 1.6 cells transfected with the indicated plasmids in response to Sendai Cantell virus (20 hemagglutination units, 24 hours). E) Fold induction of luciferase activity in cells transfected with the 3xIRF-ISRE reporter plasmids in the indicated cell lines treated during 24 hours with 500 units/ml of the corresponding species-specific IFN-α. F) Luciferase activity fold induction in murine cell lines transfected with the 3xIRF-ISRE reporter plasmid and treated with 500 units/ml murine IFN-α, 500 units/ml murine IFN-β, 50 μg/ml poly I:C or 1 μg/ml poly I:C mixed with 250 μg/ml DEAE-Dextran for 24 hours. G) Time course of luciferase activity in HuH-7 cells transfected with the 3xIRF-ISRE-luc reporter plasmids and treated with 500 units/ml of human IFN-α. These data are from one experiment representative of four. *** p<0.001, ns: not significant. TK: Thymidine kinase.

Although several plasmids containing ISRE-driven reporter elements have been generated, few studies have undertaken the task of improving such reporter plasmids and analyzing their possible use for the *in vivo* monitoring of IFN-I signature. We aimed to build an artificial optimized DNA element that responds to IFN-β induction and type I IFN signaling pathways. We hypothesized that a promoter containing such an element controlling expression of the luciferase reporter gene should be able to monitor IFN-I signature *in vivo*. In order to generate the reporter system, the IRF-ISRE enhancing element was fused to a minimal human MxA promoter. A series of reporter plasmids bearing different numbers of IRF-ISRE repeats was obtained (Fig 1A). These constructs were transfected in HuH-7 cells together with the pRL-TK plasmid in order to normalize specific firefly luciferase reporter activity. Cells were then stimulated with 500 units/ml of human IFN-α for 24 hours. A plasmid containing a tandem of the previously described ISRE sequence was included for comparison. The new IRF-ISRE sequence was more efficient than the ISRE in the response to IFN-α, even when only one repeat of this sequence was included in the plasmid (Fig 1B). Addition of multiple repeats of this element increased the potency of the reporter plasmid. The promoter containing three elements (3xIRF-ISRE) was chosen for further characterization because it showed the maximal potency without increasing baseline activity (Fig 1C). This construct also responds to the IFN-β induction pathway stimulation, such as Sendai Cantell virus infection in Hepa 1.6 cells [22] (Fig 1D). Furthermore, the 3xIRF-ISRE sequence can be induced by IFN-α in human (HuH-7 and HepG2) and mouse (Hepa 1.6 and B16.OVA) cell lines (Fig 1E) and is able to respond to different IFN-β or IFN-I signaling pathway stimuli such as IFN-α, IFN-β, Poly I:C and Poly I:C plus DEAE (Fig 1F). The kinetics of luciferase activity in response to IFN-α *in vitro* show stimulation 24 hours after induction and progressive decay, returning to baseline three days after stimulation (Fig 1G).

### *In vivo* characterization of 3xIRF-ISRE-Luc reporter plasmid delivered to mouse liver by hydrodynamic injection

**Fig 2:**
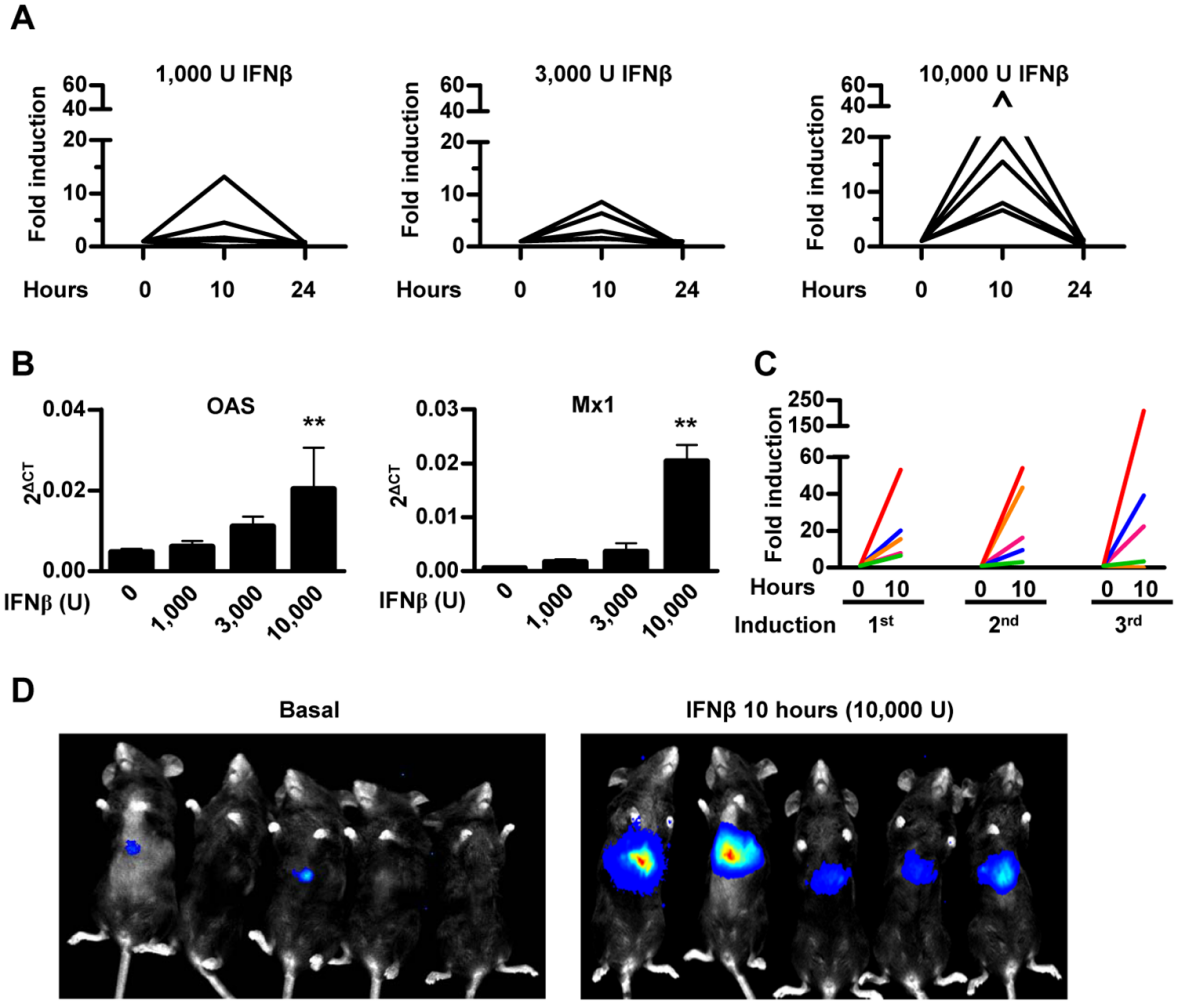
*In vivo* characterization of 3xIRF-ISRE-Luc reporter plasmid delivered to mouse liver by hydrodynamic injection. A) Mice received a hydrodynamic injection with 20 μg of 3xIRF-ISRE-Luc reporter plasmid through the tail vein. Once luciferase activity stabilized (one month after injection), mice were treated intraperitoneally with the indicated doses of murine IFN-β. Light emission was quantified by BLI 10 and 24 hours after treatment. Values correspond to fold luciferase activity, using baseline (pre-induction) activity as a reference. B) Quantitative RT-PCR of *OAS* and *Mx1* genes in peripheral blood lymphocytes of animals treated for 24 hours with different doses of recombinant murine IFN-β. C) Reporter activity re-induction in mice determined every week by intraperitoneally administration of 10,000 units of IFN-β. Each line represents an individual mouse. D) Representative BLI images of mice before and 10 hours after administration of 10,000 U of murine IFN-β. These data are from one experiment representative of three.** p<0.01 *vs* 3,000, 1,000 and 0 IFN-β units.

The ability of the 3xIRF-ISRE-Luc reporter plasmid to respond to murine IFN-β *in vivo* was analyzed using hydrodynamic plasmid injection through the tail vein in mice. Using this method of gene delivery, plasmids penetrate hepatocytes in high amounts. An intense initiation of transgene expression soon after injection is usually followed by a rapid reduction with further stabilization 3–4 weeks later [23]. Once the luciferase signal was stable (four weeks after hydrodynamic injection, data not shown), animals in three different groups received 1,000, 3,000 or 10,000 units of murine IFN-β intraperitoneally. *In vivo* bioluminescent imaging after administration of the substrate D-luciferin revealed an induction of reporter gene expression 10 hours after murine IFN-β treatment. We observed that, at the maximum dose of IFN-β (10,000 U) all animals responded consistently, while at doses of 1,000 and 3,000 U of IFN-β some of the animals showed no increase in transgene expression. Luciferase activity returned to baseline levels after 24 hours (Fig 2A). Activation of the IFN-I pathway was verified by quantifying mRNA expression of ISGs such as *OAS* and *Mx1* in peripheral blood leukocytes 10 hours after treatment (Fig 2B). A good correlation was observed between activation of IFN-β downstream genes and BLI, supporting the adequate specificity and sensitivity of the reporter system. Luciferase expression was consistently re-induced every week for at least 3 times by the administration of 10,000 units of murine IFN-β (Fig 2C). As expected, light emission was localized mainly in the liver (Fig 2D)

### *In vivo* activity of AAV8-3xIRF-ISRE-Luc in response to IFN-agonist

**Fig 3:**
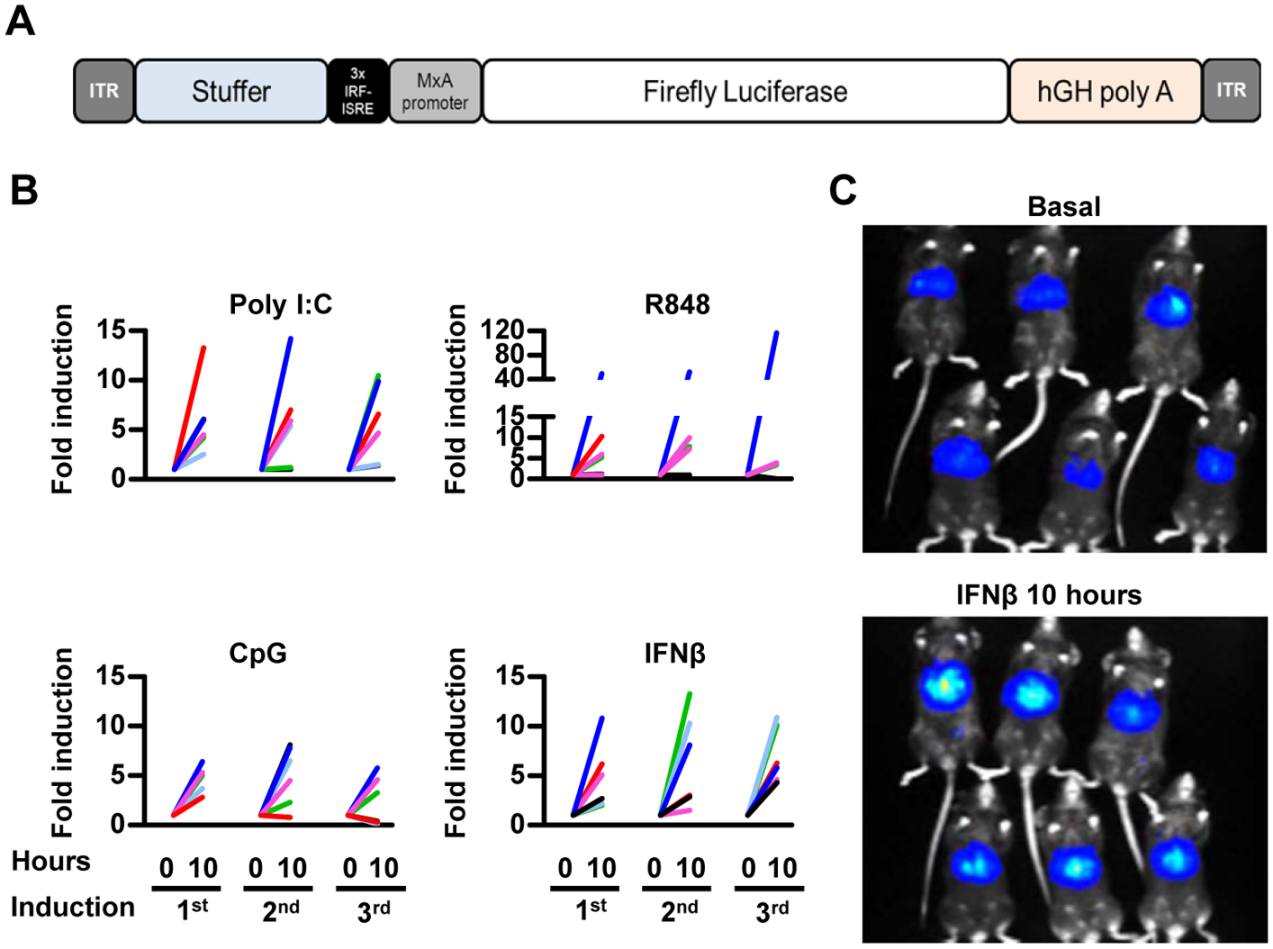
*In vivo* activity of AAV8-3xIRF-ISRE-Luc in response to IFN-agonist. A) Schematic representation of the AAV8-3xIRF-ISRE-Luc vector (not drawn to scale). B) Mice were inoculated iv with 3x10^10^ vg of the vector. Two weeks later, animals were divided in different groups and received the following iv stimuli: poly I:C, CpG DNA, Imiquimod (R848) or murine IFN-β. Treatments were repeated weekly for 3 weeks. Luciferase activity was quantified by BLI. Each line represents the fold luciferase activity of each individual mouse. C) Representative BLI images of AAV8-3xIRF-ISRE-Luciferase transduced mice before and 10 hours after murine IFN-β administration. These data are from one experiment representative of two.

We constructed a rAAV8 vector carrying the 3xIRF-ISRE-Luc cassette (Fig 3A). A dose of 3x10^10^ viral genomes/mouse of the AAV8-3xIRF-ISRE-Luc vector was injected intravenously in C57BL/6 mice. Baseline luciferase activity was measured until it stabilized approximately 2 weeks after injection, as expected for this type of vector [19]. We detected no elevations of type I IFN protein in sera from mice at days 1 and 7 after AAV8-3xIRF-ISRE-Luc vector inoculation (data not shown). Four groups of mice received different IFN-agonists: poly I:C, CpG DNA, Imiquimod (R848) or 10,000 units of murine IFN-β. Administration of agonists was repeated every week for 3 times. In all cases, an increase in luciferase signal was observed following each round of stimulation (Fig 3B). Light emission was localized mainly in the liver (Fig 3C).

### *In vivo* activity of AAV8-3xIRF-ISRE-Luc in mice in response to intravenous NDV-GFP administration

**Fig 4:**
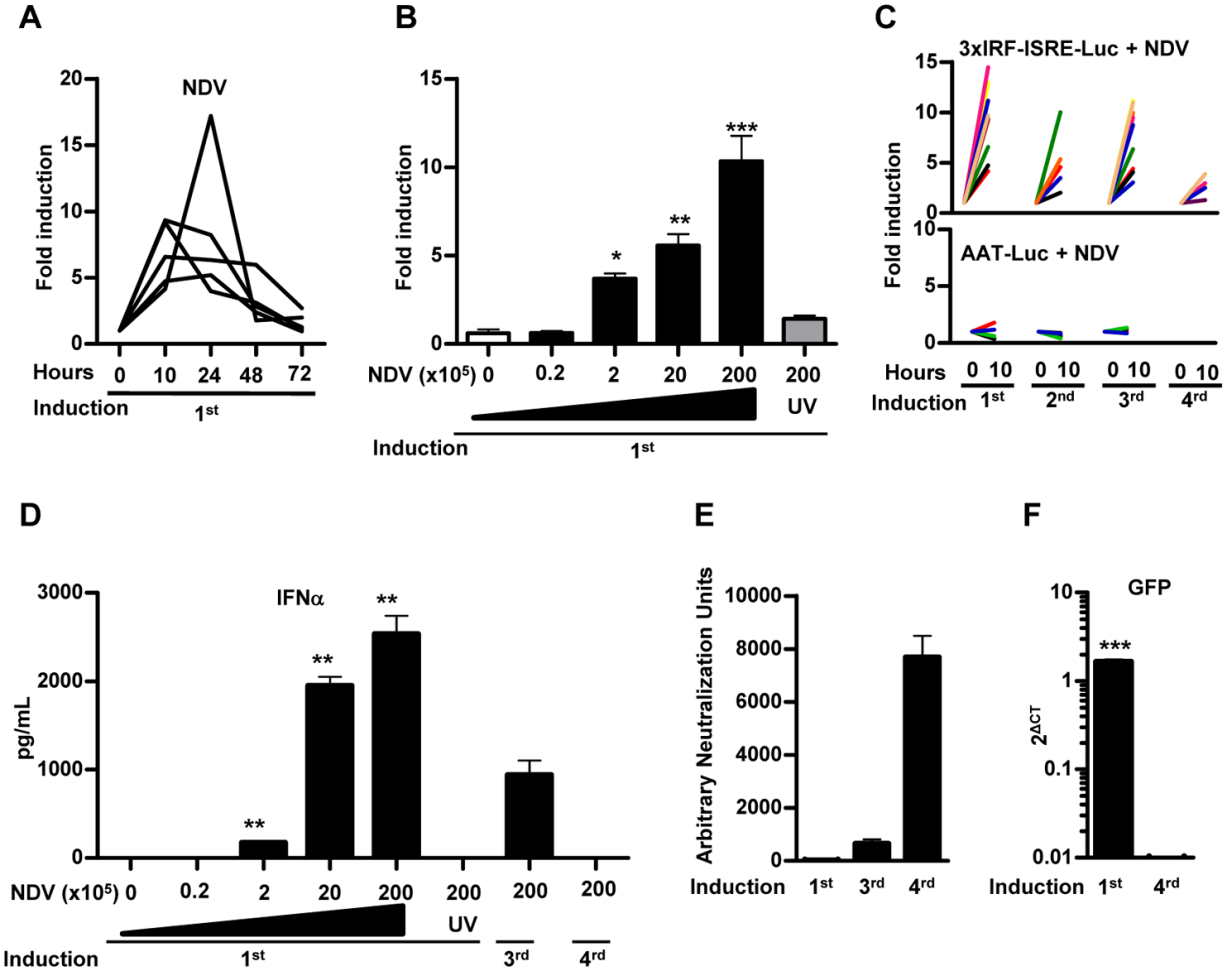
*In vivo* activity of AAV8-3xIRF-ISRE-Luc in mice in response to intravenous NDV-F3AA-GFP LaSota administration. Mice were inoculated iv with 3x10^10^ vg of the AAV8-3xIRF-ISRE-Luc vector or a control vector AAV8-AAT-Luc, in which luciferase expression is controlled by a constitutive liver-specific promoter. Two weeks later, animals started to receive iv administrations of NDV-F3AA-GFP LaSota virus at different doses and schedules A) *In vivo* luciferase activity stimulation in the AAV8-3xIRF-ISRE-Luc transduced animals at 10, 24, 48 and 72 hours after iv administration of the NDV virus at 2x10^7^ iu. Each line represents an individual mouse. B) Mice transduced with AAV8-3xIRF-ISRE-Luc received the indicated doses of NDV-F3AA-GFP LaSota virus or UV-inactivated virus (UV), and the stimulation of luciferase activity was determined 10 hours later (indicated as average fold induction for each group). C) Mice transduced with AAV8-3xIRF-ISRE-Luc (upper panel) or AAV8-AAT-Luc (lower panel) received repeated iv inoculations of 2x10^7^ iu NDV one week apart. Stimulation of luciferase activity was determined 10 hours after every NDV injection. Each line represents an individual mouse. D) Concentration of IFN-α in the serum of mice, determined by ELISA 24 hours after each NDV administration E) NDV neutralizing antibodies in serum of mice, determined one day after each round of NDV administration. F) A sub-group of mice was sacrificed after the first or fourth NDV-GFP administration, and expression of virally encoded GFP was determined by qRT-PCR in liver samples. These data are from one experiment representative of three.

It has been previously shown that the intravenous administration of NDV induces IFN-I in the mouse liver [10]. In order to validate the activity of our reporter vector *in vivo* in response to NDV, mice were treated with AAV8-3xIRF-ISRE-Luc and then received an iv administration of NDV-F3AA-GFP LaSota (2x10^7^ iu). A consistent and specific stimulation of luciferase signal was observed as early as 10 hours after virus exposure and the activity returned to baseline levels 48–72 hours after NDV infection (Fig 4A). Further experiments demonstrated a dose-dependent activation of the reporter system in response to NDV (Fig 4B), whereas inactivation of the virus by freeze/thaw and UV irradiation abolished the increase in luciferase signal. Luciferase expression was consistently re-induced every week for at least 3 times after administration of the same dose of NDV (Fig 4C). No increase in luciferase signal was observed when the same dose of NDV-F3AA-GFP LaSota virus was administered to mice previously transduced with an AAV8 vector expressing luciferase under the control of the constitutive liver-specific alpha-1 anti-trypsin promoter (AAV8-AAT-Luc) [24] (Fig 4C). Interestingly, repeated activation of the AAV8-3xIRF-ISRE-Luc reporter during the first month correlated with the stimulation of the IFN-I response evidenced by the increase in serum IFN-α after each viral infection (Fig 4D). The reporter activation was drastically diminished when the NDV virus was administered again at later times (Fig 4C), coinciding with the lack of IFN-I elevation in serum after 24 hours (Fig 4D), the appearance of high titers of anti-NDV neutralizing antibodies (Fig 4E) and the inability to detect the virus infection in the liver (Fig 4F). These results indicate that the AAV8-3xIRF-ISRE-Luc reporter is able to detect specifically the activation of IFN-I responses in the liver resulting from infection by NDV.

### *In vivo* activity of AAV8-3xIRF-ISRE-Luc in hamster in response to intravenous NDV-GFP administration

**Fig 5:**
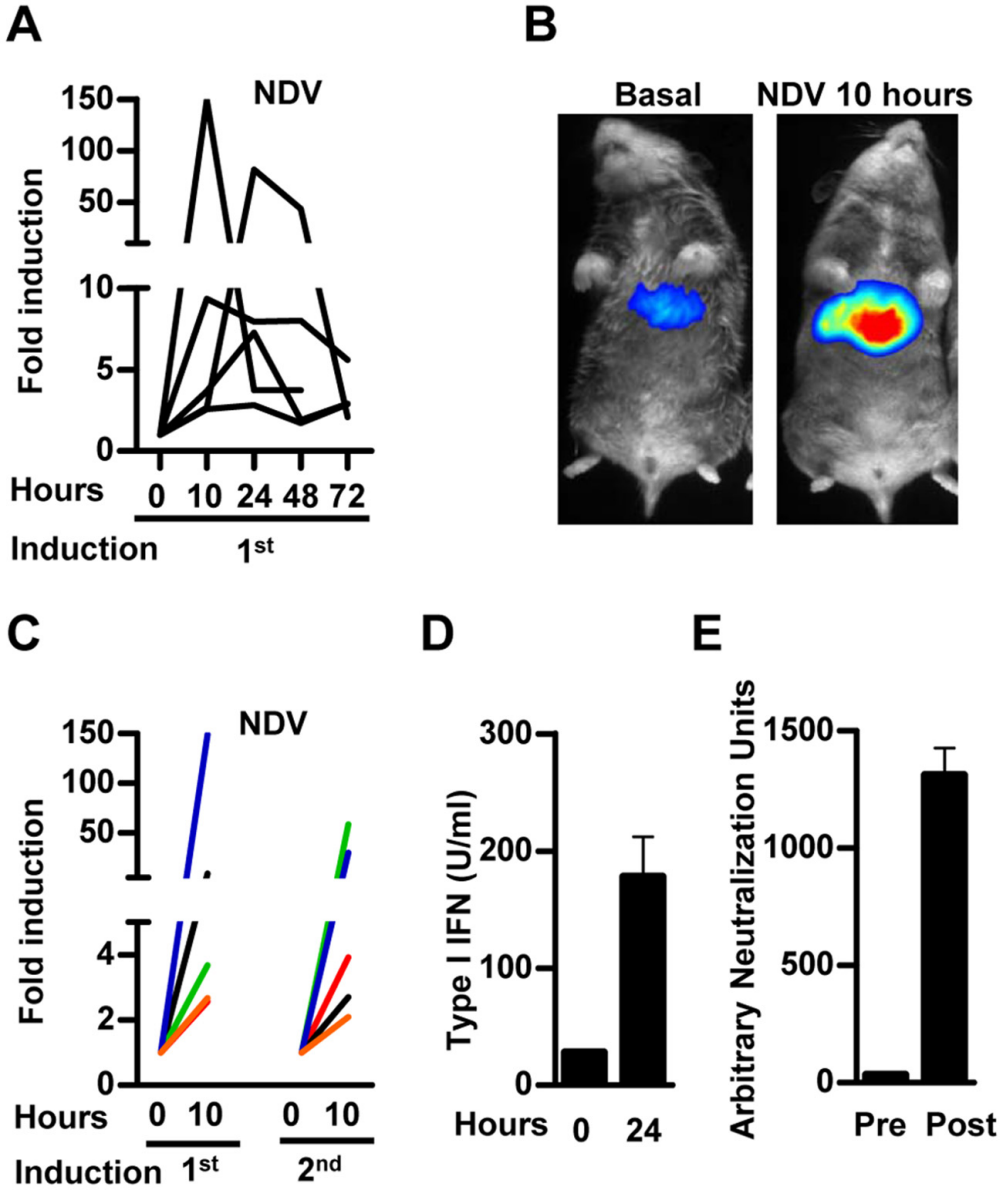
*In vivo* activity of AAV8-3xIRF-ISRE-Luc in hamster in response to intravenous NDV-F3AA-GFP LaSota administration. Syrian hamsters were inoculated iv with 1x10^11^ vg of the AAV8-3xIRF-ISRE-Luc vector. Three weeks later, animals received an iv administration of 1x10^9^ iu NDV-F3AA-GFP LaSota. A) *In vivo* luciferase activity was monitored at 10, 24, 48 and 72 hours after iv administration of the NDV virus. Each line represents an individual hamster. B) Image of a representative hamster before (basal) and 10 hours after the first NDV administration. C) Luciferase activity stimulation in hamsters receiving two doses of NDV-F3AA-GFP LaSota 6 weeks apart one from the other. D) Type I IFN activity in serum of hamsters before and 24 hours after second NDV administration, measured by bio-assay. E) NDV neutralizing antibodies in serum of hamsters before and 6 weeks after the first NDV administration. These data are from one experiment representative of two.

In order to study the ability of AAV8-3xIRF-ISRE-Luc reporter vector to detect IFN-I signature in animals other than mice, we used Syrian hamsters as a model. These animals are suitable for BLI [25] and have been used to study different viral infections due to their broad permissiveness [26–30]. After iv AAV8-3xIRF-ISRE-Luc vector administration, baseline luciferase activity *in vivo* was monitored until stabilization. As observed previously in mice, the main luciferase signal in hamsters originated from the liver (data not shown), which indicates a preferential hepatic transduction of the AAV8 vector. Intravenous administration of NDV-F3AA-GFP LaSota induced the luciferase reporter activity in the liver (Fig 5A and 5B), with kinetics similar to those observed in mice treated with the same virus (Fig 5A). A second stimulation of the reporter in response to NDV injection was observed 6 weeks later (Fig 5C). This is consistent with a peak of IFN-I in serum (Fig 5D). Under these experimental conditions, the titer of anti-NDV neutralizing antibodies is still moderate in hamsters (Fig 5E), which may explain the preservation of the IFN-I response.

### *In vivo* reporter activity of AAV8-3xIRF-ISRE-Luc in the respiratory tract

**Fig 6:**
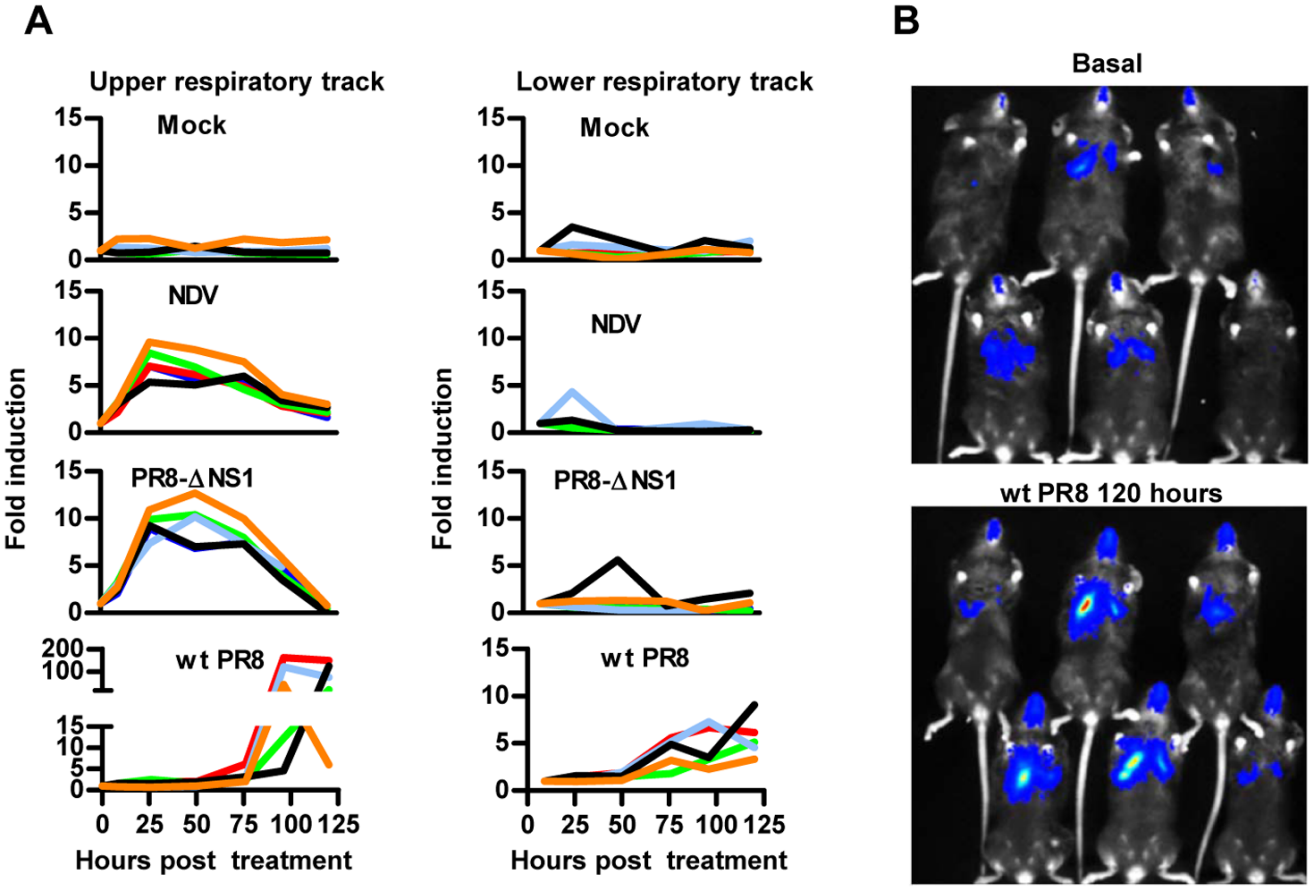
*In vivo* reporter activity of AAV8-3xIRF-ISRE-Luc in the respiratory tract. Mice received an intranasal instillation of AAV8-3xIRF-ISRE-Luc (1x10^11^ viral genomes/mouse) and were then divided in 4 groups, according to the following intranasal stimuli: Saline solution (Mock); 2x107 iu NDV-F3AA-GFP LaSota; 2x10^7^ iu influenza A/PR8/34-ΔNS1 or 2x10^2^ iu Wt A/PR8/34. A) Luciferase activity was measured by BLI in the upper and lower respiratory tract at the indicated times. B) Representative images of mice before (baseline) and 120 hours after Wt PR8 infection. Each line represents an individual mouse. These data are from one experiment representative of two.

Although not very efficiently, AAV8 can also transduce the respiratory tract after intranasal instillation. We employed this route to test if our reporter vector could deliver the 3xIRF-ISRE-driven luciferase reporter in the respiratory tract. This should allow detection of IFN-I induction elicited by airway infection with viruses displaying different pathogenicity. These viruses include a non-pathogenic virus such as NDV-F3AA-GFP LaSota (10^7^ iu/mouse) or mouse-adapted influenza viruses: the attenuated mutant A/PR8/34-ΔNS1 (10^7^ iu/mouse) and the corresponding wild type (Wt) A/PR8/34 (2x10^2^ iu /mouse). Our results indicate that the mouse immune system reacted against NDV, as shown by a potent activation of the reporter element circumscribed to the upper respiratory tract, which lasted approximately 3 days (Fig 6A). A similar result was observed after infection with A/PR8/34-ΔNS1, which was well-tolerated despite the high dose used (Fig 6A). This is compatible with an apparent localized infection of the upper respiratory tract and efficient limitation of viral spread by the strong innate immune response. To allow monitorization of Wt A/PR8/34 infection over time, a sublethal dose of the virus was intranasally inoculated in mice. In contrast to NDV or the attenuated A/PR8/34-ΔNS1 influenza virus, a robust IFN-I signature was detected in the upper and lower respiratory tracts of animals, starting 3–4 days after infection (Fig 6A and 6B). This result is consistent with active replication of the Wt A/PR8/34 virus in the lung as well as the activation of a strong inflammatory process which is type I IFN-dependent as previously reported [31,32]. The effect of virus replication on the reporter activation in the mouse upper and lower respiratory tracts can be clearly visualized 120 hours after infection (Fig 6B).

## Discussion

We describe here a novel technique to monitor IFN-I *in vivo* without the need for animal transgenesis by the administration of a non-replicating AAV vector. We demonstrate the utility of this system *in vivo* by characterizing different factors capable of enhancing IFN-I expression or signaling. A vector such as AAV8-3xIRF-ISRE-Luc is a novel and valid biotechnological tool to follow IFN-I signature in mice and hamsters and most likely in many more animal species since the IRF and ISRE promoter sequences are conserved across vertebrates [33].

In the present study, we have been able to monitor IFN-I signature after administration of NDV-GFP, a chicken-adapted virus, with limited replication in mammals [34]. A detailed characterization in mice indicated that the activation of the reporter system in the liver was dose-dependent and correlated with the presence of IFN-α in serum (Fig 4). Interestingly, the system was able to detect repeated activations of the IFN-I response upon NDV re-administration, until the titer of neutralizing antibodies was high enough to block liver infection. At this point, a residual reporter signal was present in the absence of detectable IFN-α in serum. This may reflect a higher sensitivity of the AAV8-3xIRF-ISRE-Luc vector compared to the IFN-α ELISA or the detection of NDV-encoded GFP by qRT-PCR. Alternatively, it is possible that the IFN-I response is activated in these circumstances in the absence of liver infection. Although the mechanism is still speculative, one possibility is that antibody-neutralized NDV immune complex could be uptaken by a particular phagocytic cell in the liver. This neutralized virus genetic material could be detected and trigger type I IFN in a process similar to the one that triggers the nucleic acid-antibody immune-complexes responsible for type I IFN stimulation in systemic lupus erythematosus [35]. In addition, although not described for NDV, it has been proposed that some virus infections, specifically in the entry process, could be enhanced by virus opsonization [36]. Alternatively, a small fraction of NDV could escape from antibody-driven phagocytosis and be detected by previously described FcγR negative monocytes (CD64- or CD64 low) which could trigger the production of type I IFN [37]. Further work will determine if the same pattern of response is observed in different species. We provide evidence that the reporter can be re-induced by NDV infection in Syrian hamsters when the titer of antibodies is still relatively low and there is new peak of IFN-I in serum, but long-term follow-up in this and other animal models will expand our knowledge about innate immune response against this virus.

The study performed with the influenza viruses, A/PR8/34-ΔNS1 and Wt A/PR8/34 highlights the relevance and usefulness of the reporter vector presented here and its possible adaptation to different organs, in this case the respiratory tract. Our results indicate that influenza induces a mild and early IFN-I response capable of controlling viral infection when the virus lacks the NS1 protein, whereas this response is not detected when the virus expresses NS1. In fact, the NS1-expressing virus induces a delayed but very strong IFN-I response clearly detected by the reporter system in the lower respiratory tract. This signal correlates with a robust immune response compatible with an effective replication of influenza virus in these mice. Equivalent studies can be carried out to identify new pathogenic factors related to the IFN-I response *in vivo* after infection with a variety of viruses and animal models where AAV could be employed as the delivery vector.

*In vivo* bioluminescence detection requires specialized instrumentation that may not be adapted easily to large animals. In these situations an IFN-I signature could be detected *in vivo* by replacing the luciferase gene with a soluble reporter gene easily detected in blood, such as secreted alkaline phosphatase (SEAP) [38] or others. This system could bypass the requirement for specie-specific type I IFN ELISA kits. The inducible sequence could also be moved to other viral vectors or organ-specific nanoparticles that would be useful to monitor specific activation of type I IFN upon different stimuli.

Alternatively the reporter element described here could be inserted into the genome of different nuclear DNA replicating viruses. Indeed, activation of this reporter in infected cells may be useful to study kinetics of the type I IFN response *in vivo* in the infected cell.

Finally, a potential application of this tool in the field of gene therapy would involve the use of the IFN-responsive promoter to control the expression of a therapeutic gene. Delivery of such construct in the target organ would achieve activation of transgene expression in response to endogenous IFN responses. Alternatively, recombinant type I IFN could be used as an inducer.

## Conclusions

We present here a novel tool to deliver an IFN-I responsive reporter system to the livers or the respiratory tract that could be extended to other organs by changing the vector, AAV serotype and/or the route of administration. This convenient method allows sustained monitoring of IFN-I induction *in vivo* without the need for breeding transgenic animals. Furthermore, this reporter system could be used in different animal species, in which transgenic reporters are not available, increasing the opportunities of studying IFN-I induction by a wide number of viruses or agents.
